# Supplementary material for: Trabid patient mutations impede the axonal trafficking of adenomatous polyposis coli to disrupt neurite growth
Source: eLife. 2023 Dec 15;12:RP90796. doi: 10.7554/eLife.90796 (PMC10723793; doi:10.7554/eLife.90796)
Supplement: Figure 1—source data 1. [file elife-90796-fig1-data1.zip › Figure 1-source data 1/Figure 1-source data 1.pdf]

Figure 1-source data 1

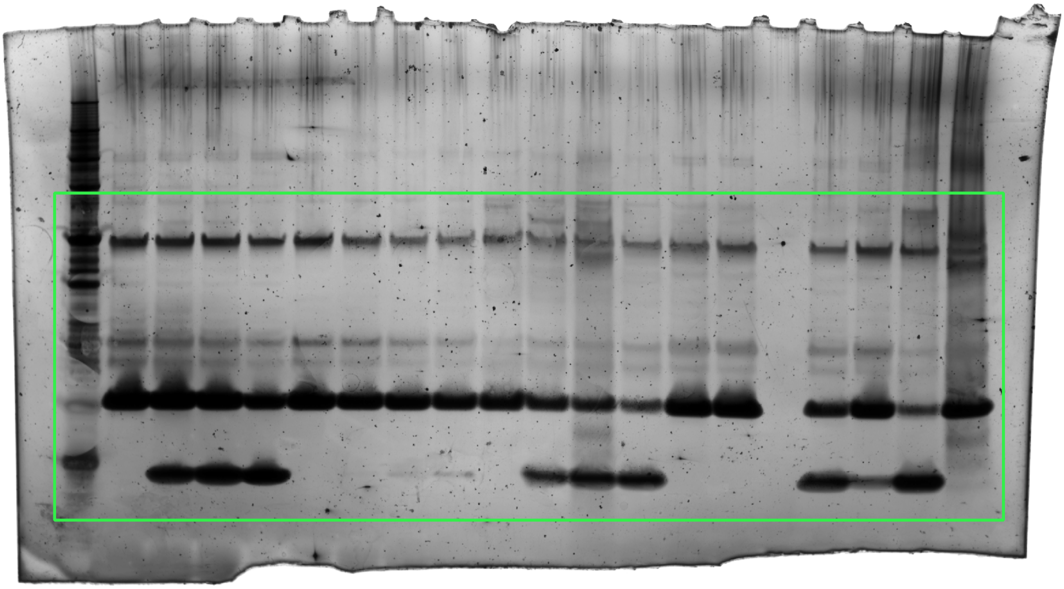

Uncropped silver-stained gel of Lys29 di-Ub DUB assay.
The region in the green box is presented as Figure 1C.

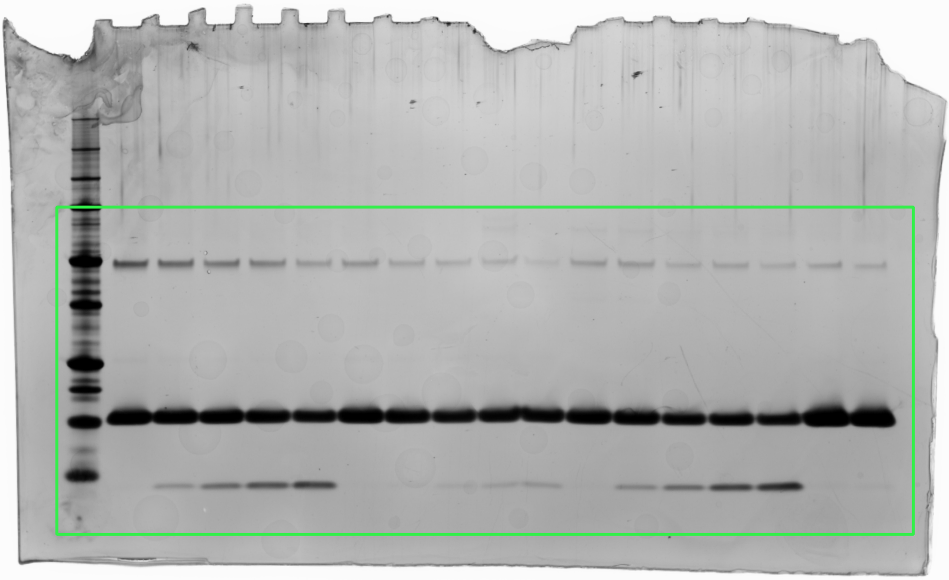

Uncropped silver-stained gel of Lys63 di-Ub DUB assay.
The region in the green box is presented as Figure 1D.
