# Supplementary figures and images for: Trabid patient mutations impede the axonal trafficking of adenomatous polyposis coli to disrupt neurite growth
Source: eLife. 2023 Dec 15;12:RP90796. doi: 10.7554/eLife.90796 (PMC10723793; doi:10.7554/eLife.90796)

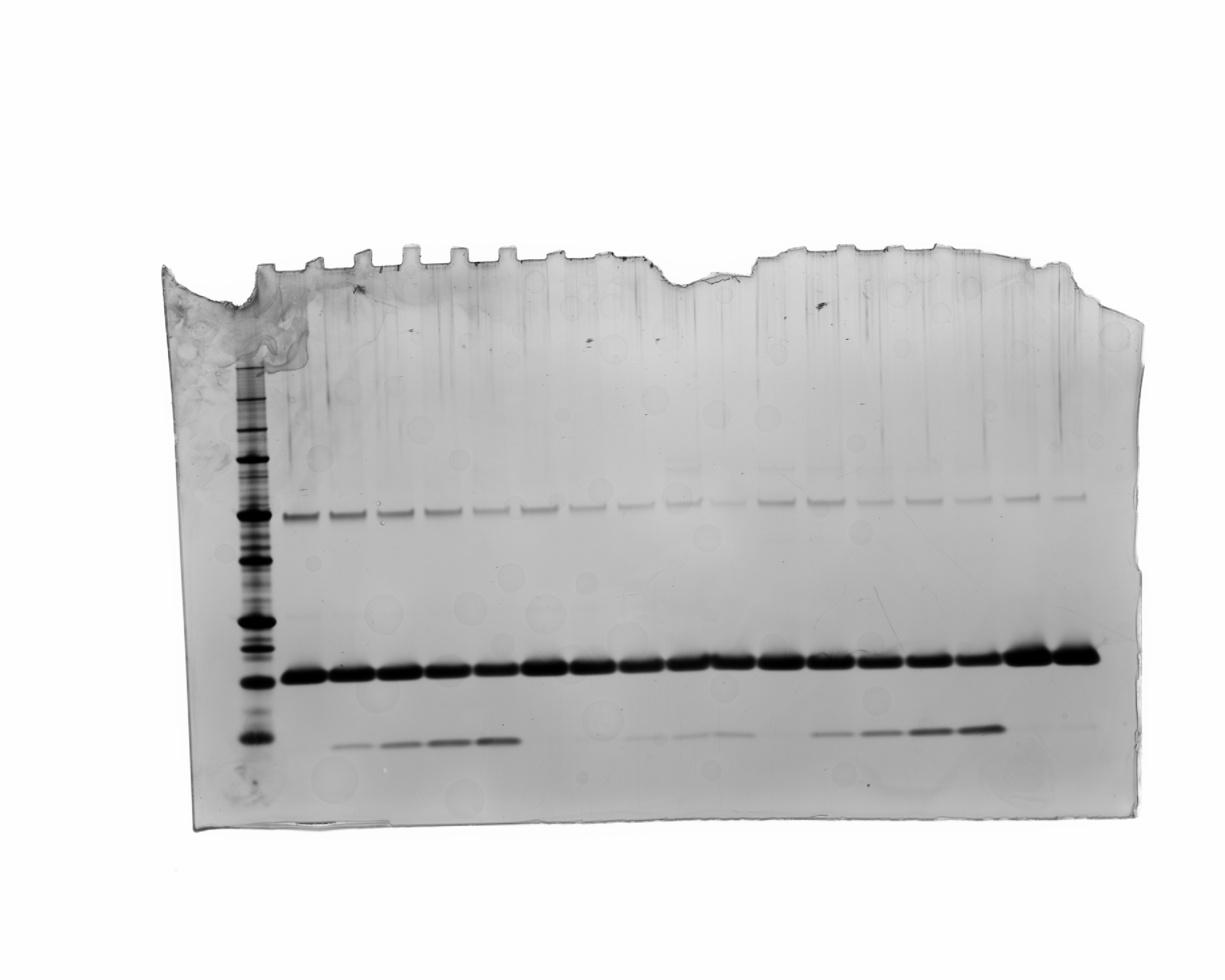

Supplement: Figure 1—source data 1. [file elife-90796-fig1-data1.zip › Figure 1-source data 1/Figure 1D uncropped_Daniel Frank.tif]

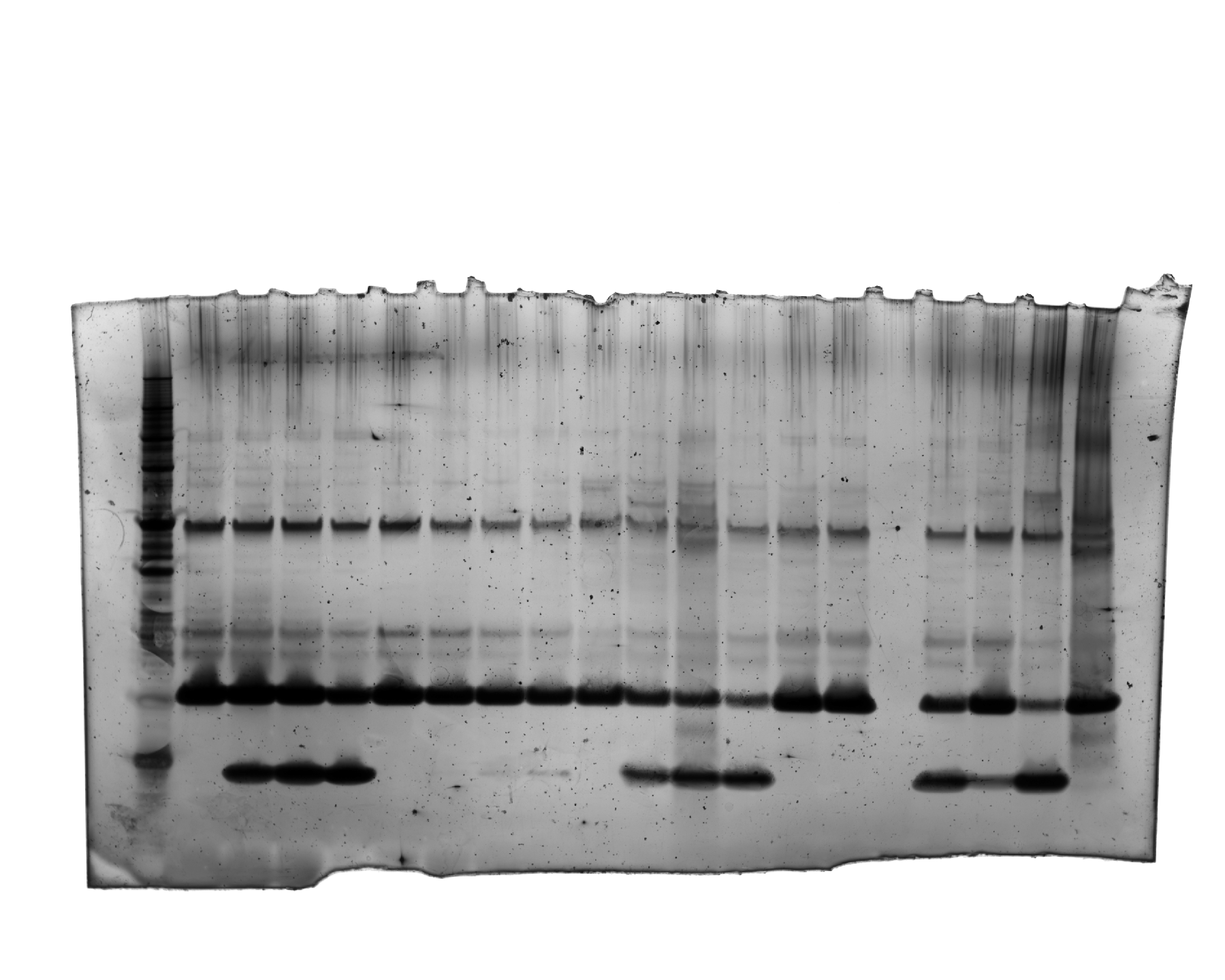

Supplement: Figure 1—source data 1. [file elife-90796-fig1-data1.zip › Figure 1-source data 1/Figure 1C uncropped_Daniel Frank.tif]

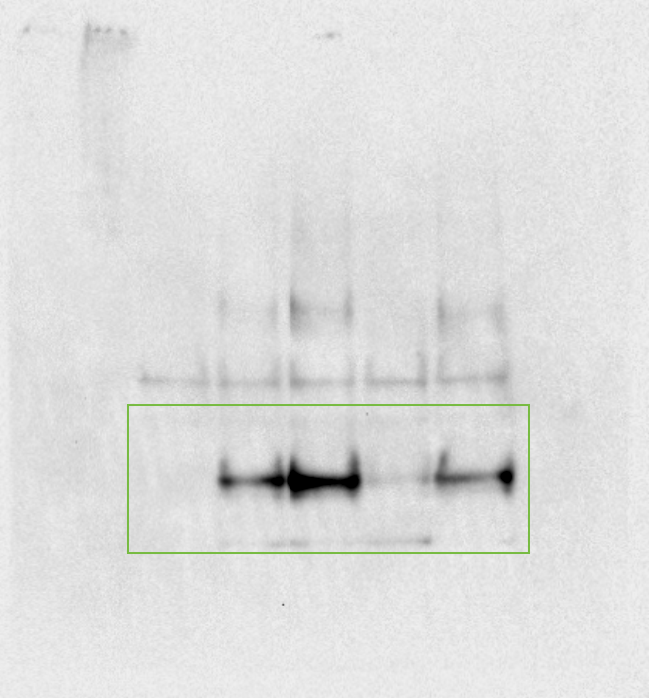

Supplement: Figure 1—source data 2. — Chemiluminescent signals on nitrocellulose blots were acquired using the ChemiDoc Imaging system (Bio-Rad). Included for each of the indicated antibody probe is the uncropped chemiluminescence image with a green box demarcating the region presented in Figure 1E. Also included is an image of the chemiluminescence signal merged with an image of the blotting membrane to visualize the positions of pre-stained molecular weight markers (M; HiMark, Invitrogen) relative to the chemiluminescent bands of interest. [file elife-90796-fig1-data2.zip › Figure 1-source data 2/FIG1E ipFLAG wbSTRIP1 uncropped_L5W 2019-02-26 12h45m35s.tif]

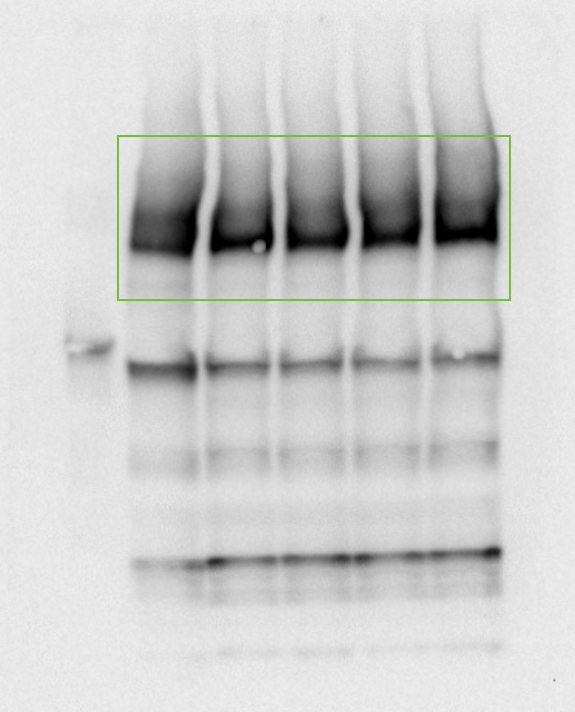

Supplement: Figure 1—source data 2. — Chemiluminescent signals on nitrocellulose blots were acquired using the ChemiDoc Imaging system (Bio-Rad). Included for each of the indicated antibody probe is the uncropped chemiluminescence image with a green box demarcating the region presented in Figure 1E. Also included is an image of the chemiluminescence signal merged with an image of the blotting membrane to visualize the positions of pre-stained molecular weight markers (M; HiMark, Invitrogen) relative to the chemiluminescent bands of interest. [file elife-90796-fig1-data2.zip › Figure 1-source data 2/FIG1E inputs wbHECTD1_L5W 2019-02-10 10h04m42s.tif]

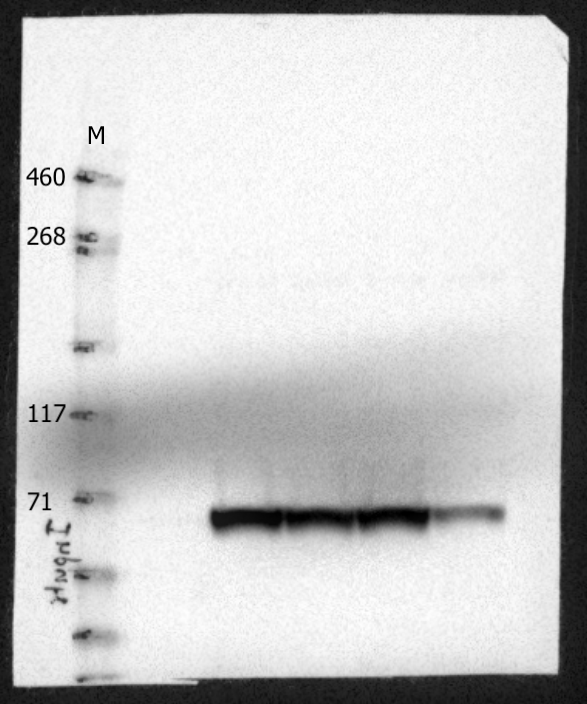

Supplement: Figure 1—source data 2. — Chemiluminescent signals on nitrocellulose blots were acquired using the ChemiDoc Imaging system (Bio-Rad). Included for each of the indicated antibody probe is the uncropped chemiluminescence image with a green box demarcating the region presented in Figure 1E. Also included is an image of the chemiluminescence signal merged with an image of the blotting membrane to visualize the positions of pre-stained molecular weight markers (M; HiMark, Invitrogen) relative to the chemiluminescent bands of interest. [file elife-90796-fig1-data2.zip › Figure 1-source data 2/FIG1E inputs wbFLAG+membrane_L5W 2019-02-09 14h28m32s+L5W 2019-02-09 14h29m39s.tif]

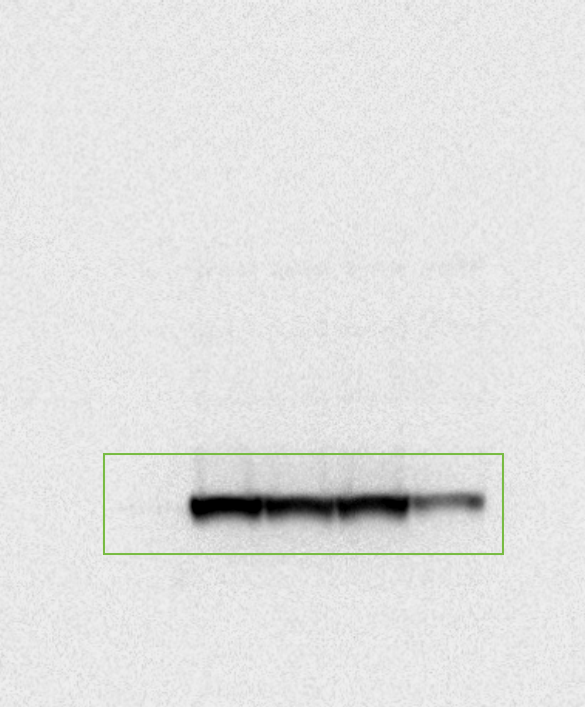

Supplement: Figure 1—source data 2. — Chemiluminescent signals on nitrocellulose blots were acquired using the ChemiDoc Imaging system (Bio-Rad). Included for each of the indicated antibody probe is the uncropped chemiluminescence image with a green box demarcating the region presented in Figure 1E. Also included is an image of the chemiluminescence signal merged with an image of the blotting membrane to visualize the positions of pre-stained molecular weight markers (M; HiMark, Invitrogen) relative to the chemiluminescent bands of interest. [file elife-90796-fig1-data2.zip › Figure 1-source data 2/FIG1E inputs wbFLAG_L5W 2019-02-09 14h28m32s.tif]

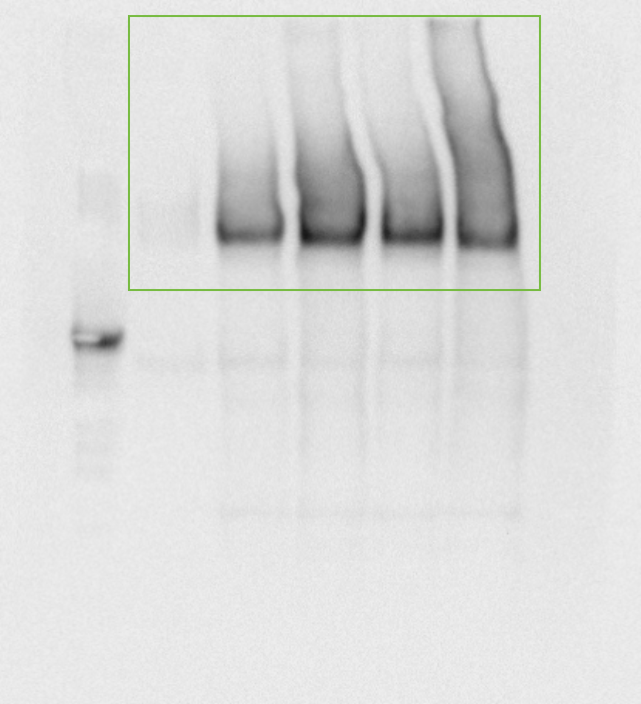

Supplement: Figure 1—source data 2. — Chemiluminescent signals on nitrocellulose blots were acquired using the ChemiDoc Imaging system (Bio-Rad). Included for each of the indicated antibody probe is the uncropped chemiluminescence image with a green box demarcating the region presented in Figure 1E. Also included is an image of the chemiluminescence signal merged with an image of the blotting membrane to visualize the positions of pre-stained molecular weight markers (M; HiMark, Invitrogen) relative to the chemiluminescent bands of interest. [file elife-90796-fig1-data2.zip › Figure 1-source data 2/FIG1E ipFLAG wbHECTD1 uncropped_L5W 2019-02-10 10h04m42s.tif]

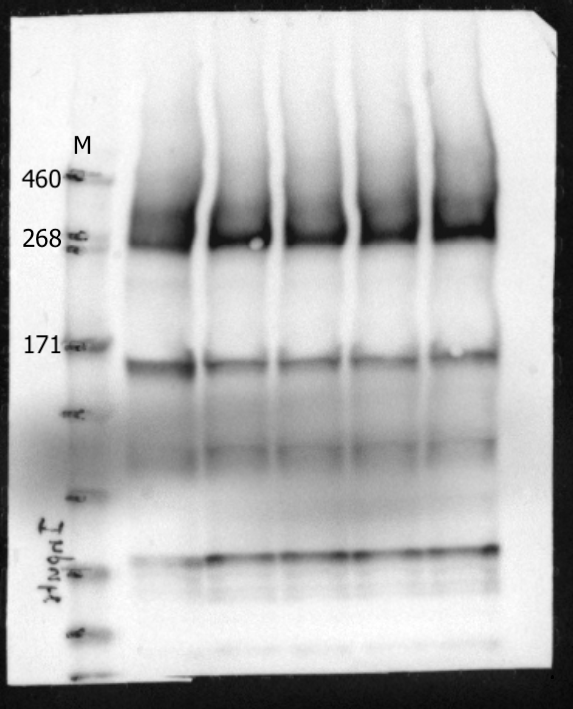

Supplement: Figure 1—source data 2. — Chemiluminescent signals on nitrocellulose blots were acquired using the ChemiDoc Imaging system (Bio-Rad). Included for each of the indicated antibody probe is the uncropped chemiluminescence image with a green box demarcating the region presented in Figure 1E. Also included is an image of the chemiluminescence signal merged with an image of the blotting membrane to visualize the positions of pre-stained molecular weight markers (M; HiMark, Invitrogen) relative to the chemiluminescent bands of interest. [file elife-90796-fig1-data2.zip › Figure 1-source data 2/FIG1E inputs wbHECTD1+membrane_L5W 2019-02-10 10h04m42s+L5W 2019-02-10 10h02m29s.tif]

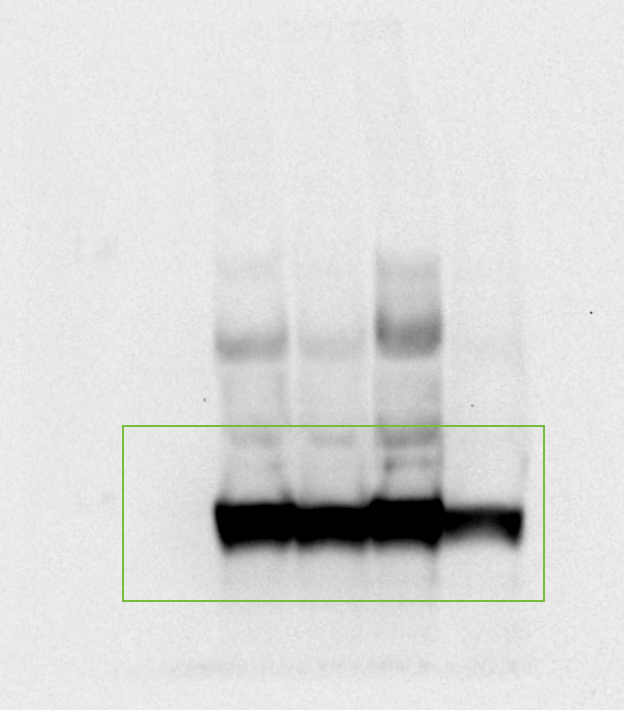

Supplement: Figure 1—source data 2. — Chemiluminescent signals on nitrocellulose blots were acquired using the ChemiDoc Imaging system (Bio-Rad). Included for each of the indicated antibody probe is the uncropped chemiluminescence image with a green box demarcating the region presented in Figure 1E. Also included is an image of the chemiluminescence signal merged with an image of the blotting membrane to visualize the positions of pre-stained molecular weight markers (M; HiMark, Invitrogen) relative to the chemiluminescent bands of interest. [file elife-90796-fig1-data2.zip › Figure 1-source data 2/FIG1E ipFLAG wbFLAG uncropped_L5W 2019-02-13 11h58m46s.tif]

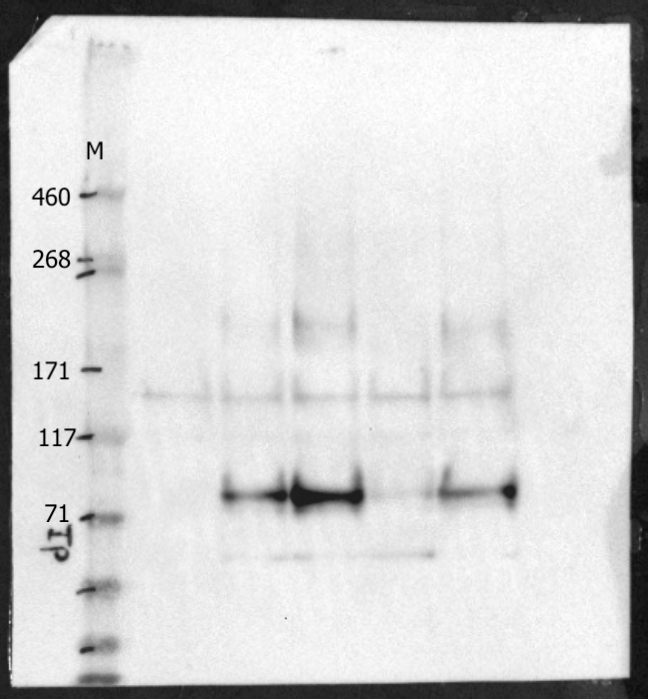

Supplement: Figure 1—source data 2. — Chemiluminescent signals on nitrocellulose blots were acquired using the ChemiDoc Imaging system (Bio-Rad). Included for each of the indicated antibody probe is the uncropped chemiluminescence image with a green box demarcating the region presented in Figure 1E. Also included is an image of the chemiluminescence signal merged with an image of the blotting membrane to visualize the positions of pre-stained molecular weight markers (M; HiMark, Invitrogen) relative to the chemiluminescent bands of interest. [file elife-90796-fig1-data2.zip › Figure 1-source data 2/FIG1E ipFLAG wbSTRIP1+membrane_L5W 2019-02-26 12h51m40s+L5W 2019-02-26 12h45m35s.tif]

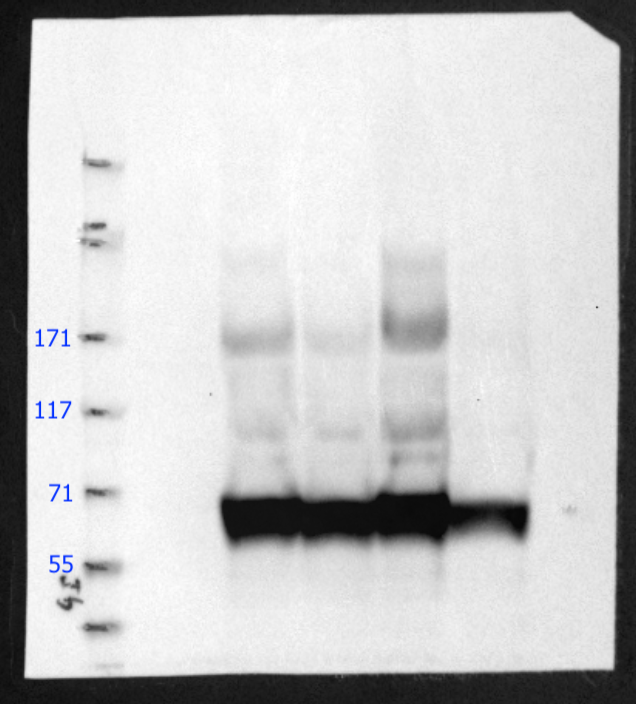

Supplement: Figure 1—source data 2. — Chemiluminescent signals on nitrocellulose blots were acquired using the ChemiDoc Imaging system (Bio-Rad). Included for each of the indicated antibody probe is the uncropped chemiluminescence image with a green box demarcating the region presented in Figure 1E. Also included is an image of the chemiluminescence signal merged with an image of the blotting membrane to visualize the positions of pre-stained molecular weight markers (M; HiMark, Invitrogen) relative to the chemiluminescent bands of interest. [file elife-90796-fig1-data2.zip › Figure 1-source data 2/FIG1E ipFLAG wbFLAG+membrane_L5W 2019-02-13 12h01m59s+L5W 2019-02-13 11h58m46s.tif]

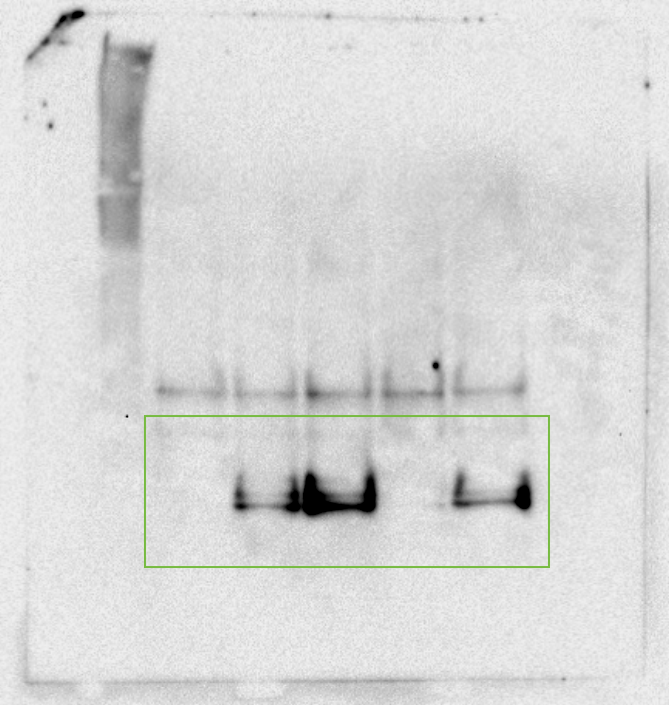

Supplement: Figure 1—source data 2. — Chemiluminescent signals on nitrocellulose blots were acquired using the ChemiDoc Imaging system (Bio-Rad). Included for each of the indicated antibody probe is the uncropped chemiluminescence image with a green box demarcating the region presented in Figure 1E. Also included is an image of the chemiluminescence signal merged with an image of the blotting membrane to visualize the positions of pre-stained molecular weight markers (M; HiMark, Invitrogen) relative to the chemiluminescent bands of interest. [file elife-90796-fig1-data2.zip › Figure 1-source data 2/FIG1E ipFLAG wbSTRN3 uncropped_L5W 2019-02-25 10h40m37s.tif]

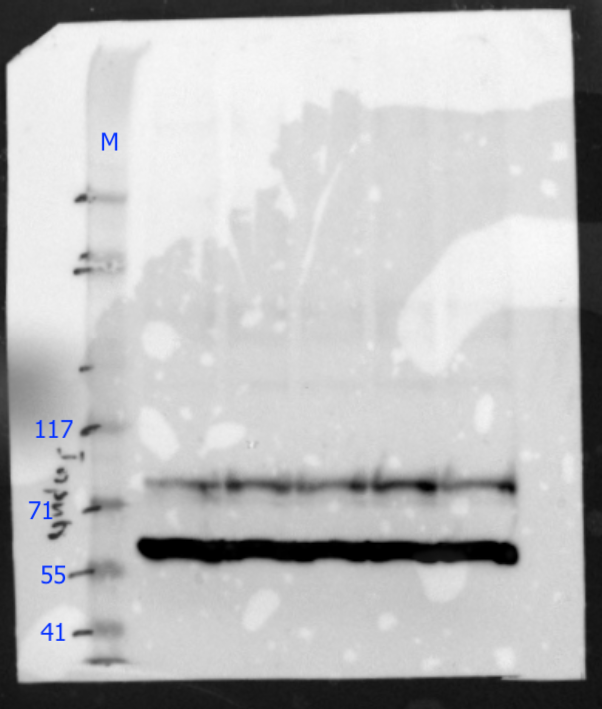

Supplement: Figure 1—source data 2. — Chemiluminescent signals on nitrocellulose blots were acquired using the ChemiDoc Imaging system (Bio-Rad). Included for each of the indicated antibody probe is the uncropped chemiluminescence image with a green box demarcating the region presented in Figure 1E. Also included is an image of the chemiluminescence signal merged with an image of the blotting membrane to visualize the positions of pre-stained molecular weight markers (M; HiMark, Invitrogen) relative to the chemiluminescent bands of interest. [file elife-90796-fig1-data2.zip › Figure 1-source data 2/FIG1E inputs wbSTRIP1+membrane_L5W 2019-02-26 12h51m40s+L5W 2019-02-26 12h43m37s.tif]

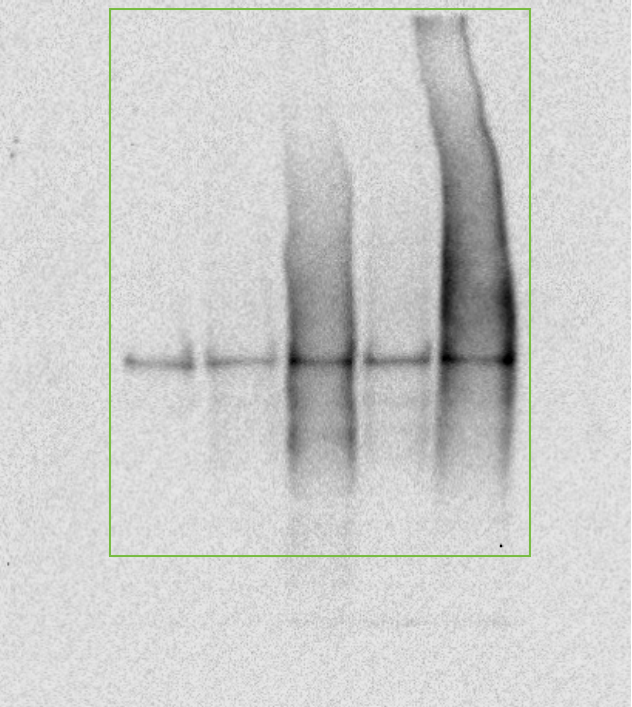

Supplement: Figure 1—source data 2. — Chemiluminescent signals on nitrocellulose blots were acquired using the ChemiDoc Imaging system (Bio-Rad). Included for each of the indicated antibody probe is the uncropped chemiluminescence image with a green box demarcating the region presented in Figure 1E. Also included is an image of the chemiluminescence signal merged with an image of the blotting membrane to visualize the positions of pre-stained molecular weight markers (M; HiMark, Invitrogen) relative to the chemiluminescent bands of interest. [file elife-90796-fig1-data2.zip › Figure 1-source data 2/FIG1E ipFLAG wbUbiquitin uncropped_L5W 2019-02-09 14h28m32s.tif]

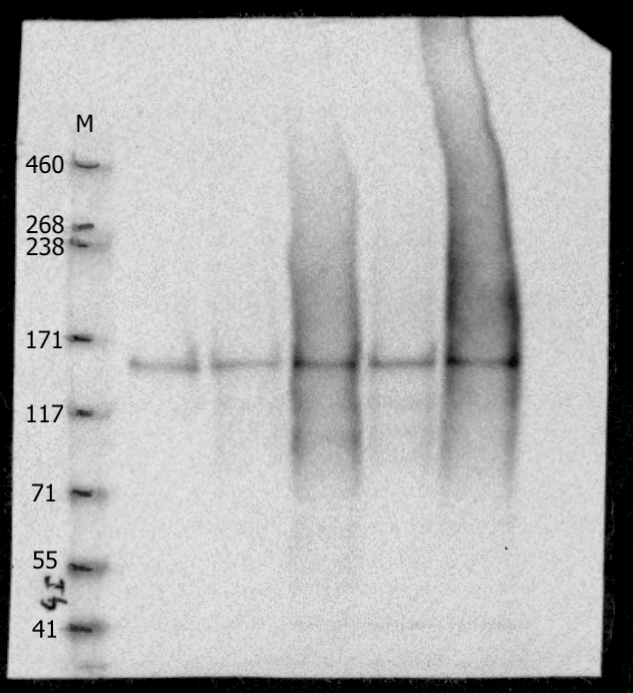

Supplement: Figure 1—source data 2. — Chemiluminescent signals on nitrocellulose blots were acquired using the ChemiDoc Imaging system (Bio-Rad). Included for each of the indicated antibody probe is the uncropped chemiluminescence image with a green box demarcating the region presented in Figure 1E. Also included is an image of the chemiluminescence signal merged with an image of the blotting membrane to visualize the positions of pre-stained molecular weight markers (M; HiMark, Invitrogen) relative to the chemiluminescent bands of interest. [file elife-90796-fig1-data2.zip › Figure 1-source data 2/FIG1E ipFLAG wbUbiquitin+membrane_L5W 2019-02-09 14h28m32s+L5W 2019-02-09 14h29m39s.tif]

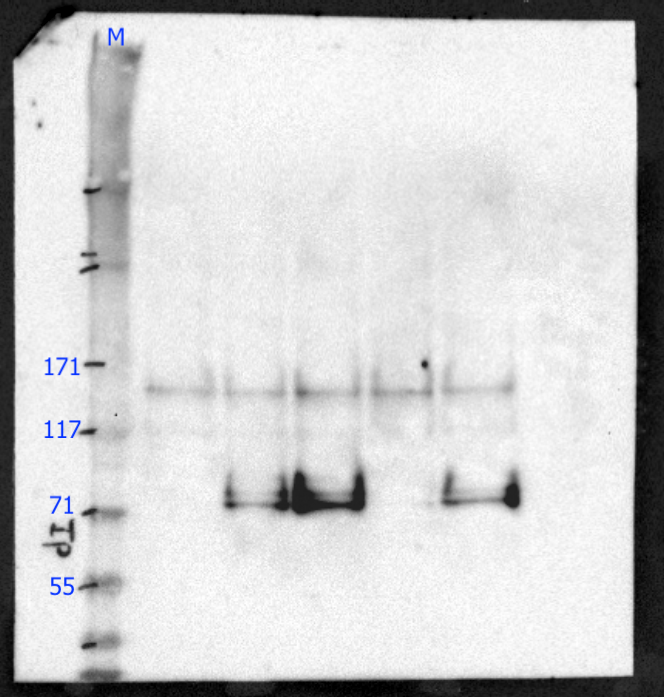

Supplement: Figure 1—source data 2. — Chemiluminescent signals on nitrocellulose blots were acquired using the ChemiDoc Imaging system (Bio-Rad). Included for each of the indicated antibody probe is the uncropped chemiluminescence image with a green box demarcating the region presented in Figure 1E. Also included is an image of the chemiluminescence signal merged with an image of the blotting membrane to visualize the positions of pre-stained molecular weight markers (M; HiMark, Invitrogen) relative to the chemiluminescent bands of interest. [file elife-90796-fig1-data2.zip › Figure 1-source data 2/FIG1E ipFLAG wbSTRN3+membrane_L5W 2019-02-25 10h40m37s+L5W 2019-02-25 10h42m25s.tif]

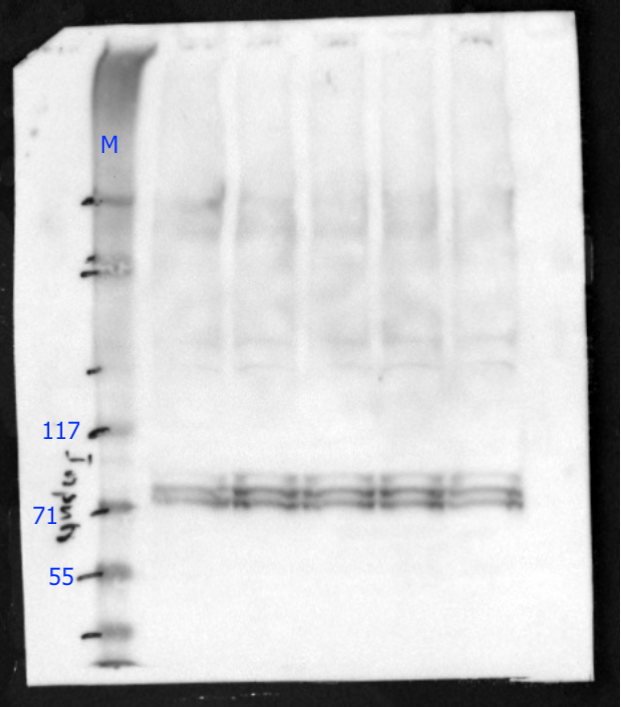

Supplement: Figure 1—source data 2. — Chemiluminescent signals on nitrocellulose blots were acquired using the ChemiDoc Imaging system (Bio-Rad). Included for each of the indicated antibody probe is the uncropped chemiluminescence image with a green box demarcating the region presented in Figure 1E. Also included is an image of the chemiluminescence signal merged with an image of the blotting membrane to visualize the positions of pre-stained molecular weight markers (M; HiMark, Invitrogen) relative to the chemiluminescent bands of interest. [file elife-90796-fig1-data2.zip › Figure 1-source data 2/FIG1E inputs wbSTRN3+membrane_L5W 2019-02-25 13h36m42s+L5W 2019-02-25 13h37m41s.tif]

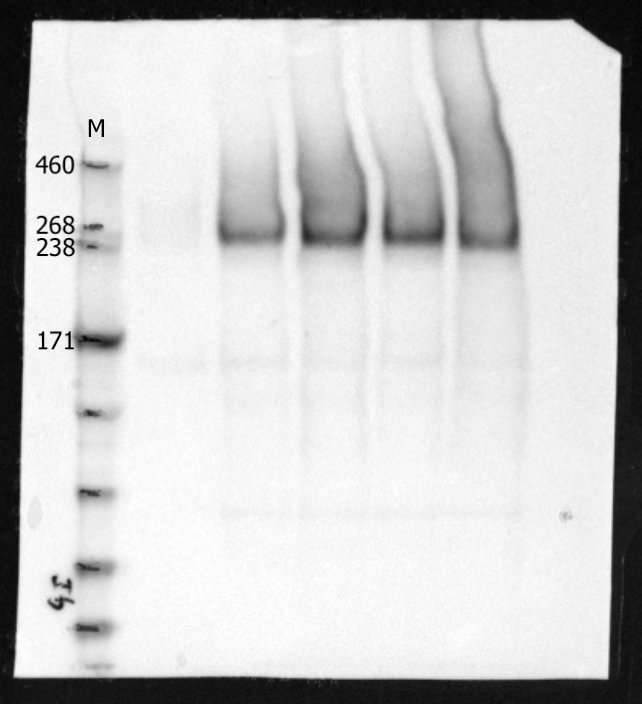

Supplement: Figure 1—source data 2. — Chemiluminescent signals on nitrocellulose blots were acquired using the ChemiDoc Imaging system (Bio-Rad). Included for each of the indicated antibody probe is the uncropped chemiluminescence image with a green box demarcating the region presented in Figure 1E. Also included is an image of the chemiluminescence signal merged with an image of the blotting membrane to visualize the positions of pre-stained molecular weight markers (M; HiMark, Invitrogen) relative to the chemiluminescent bands of interest. [file elife-90796-fig1-data2.zip › Figure 1-source data 2/FIG1E ipFLAG wbHECTD1+membrane_L5W 2019-02-10 10h04m42s+L5W 2019-02-10 10h02m29s.tif]

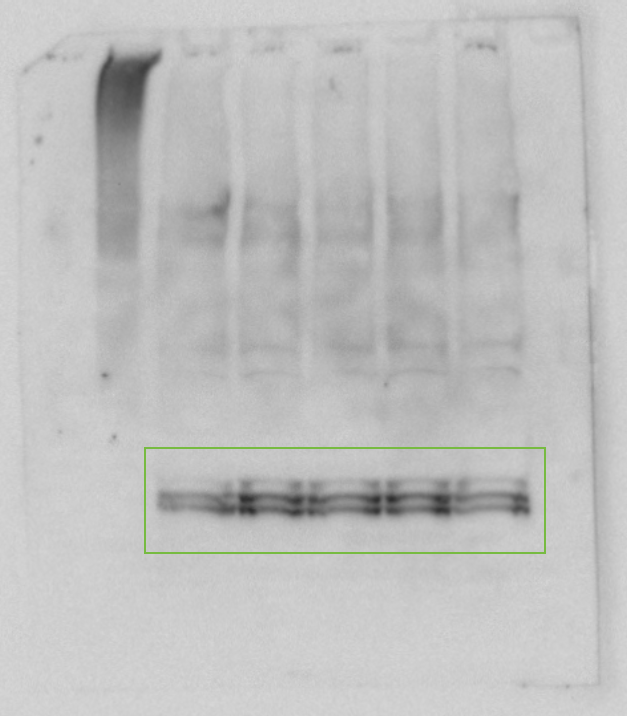

Supplement: Figure 1—source data 2. — Chemiluminescent signals on nitrocellulose blots were acquired using the ChemiDoc Imaging system (Bio-Rad). Included for each of the indicated antibody probe is the uncropped chemiluminescence image with a green box demarcating the region presented in Figure 1E. Also included is an image of the chemiluminescence signal merged with an image of the blotting membrane to visualize the positions of pre-stained molecular weight markers (M; HiMark, Invitrogen) relative to the chemiluminescent bands of interest. [file elife-90796-fig1-data2.zip › Figure 1-source data 2/FIG1E inputs wbSTRN3 uncropped_L5W 2019-02-25 13h36m42s.tif]

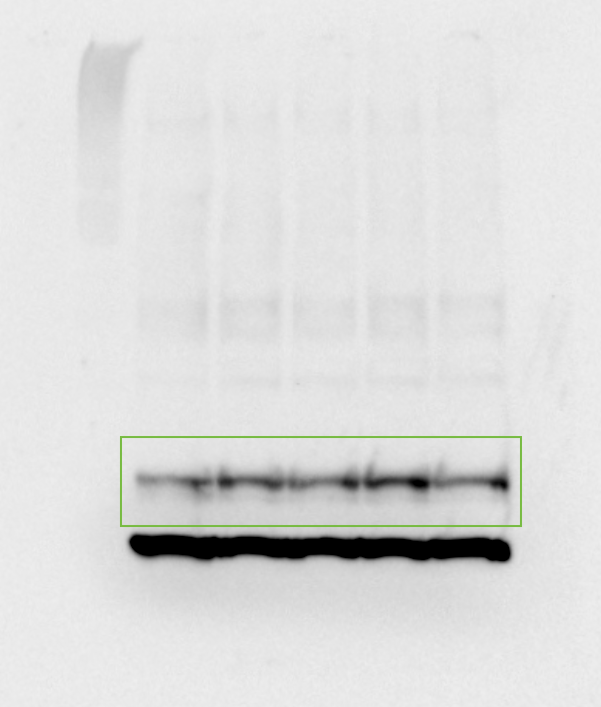

Supplement: Figure 1—source data 2. — Chemiluminescent signals on nitrocellulose blots were acquired using the ChemiDoc Imaging system (Bio-Rad). Included for each of the indicated antibody probe is the uncropped chemiluminescence image with a green box demarcating the region presented in Figure 1E. Also included is an image of the chemiluminescence signal merged with an image of the blotting membrane to visualize the positions of pre-stained molecular weight markers (M; HiMark, Invitrogen) relative to the chemiluminescent bands of interest. [file elife-90796-fig1-data2.zip › Figure 1-source data 2/FIG1E inputs wbSTRIP1 uncropped_L5W 2019-02-26 12h43m37s.tif]

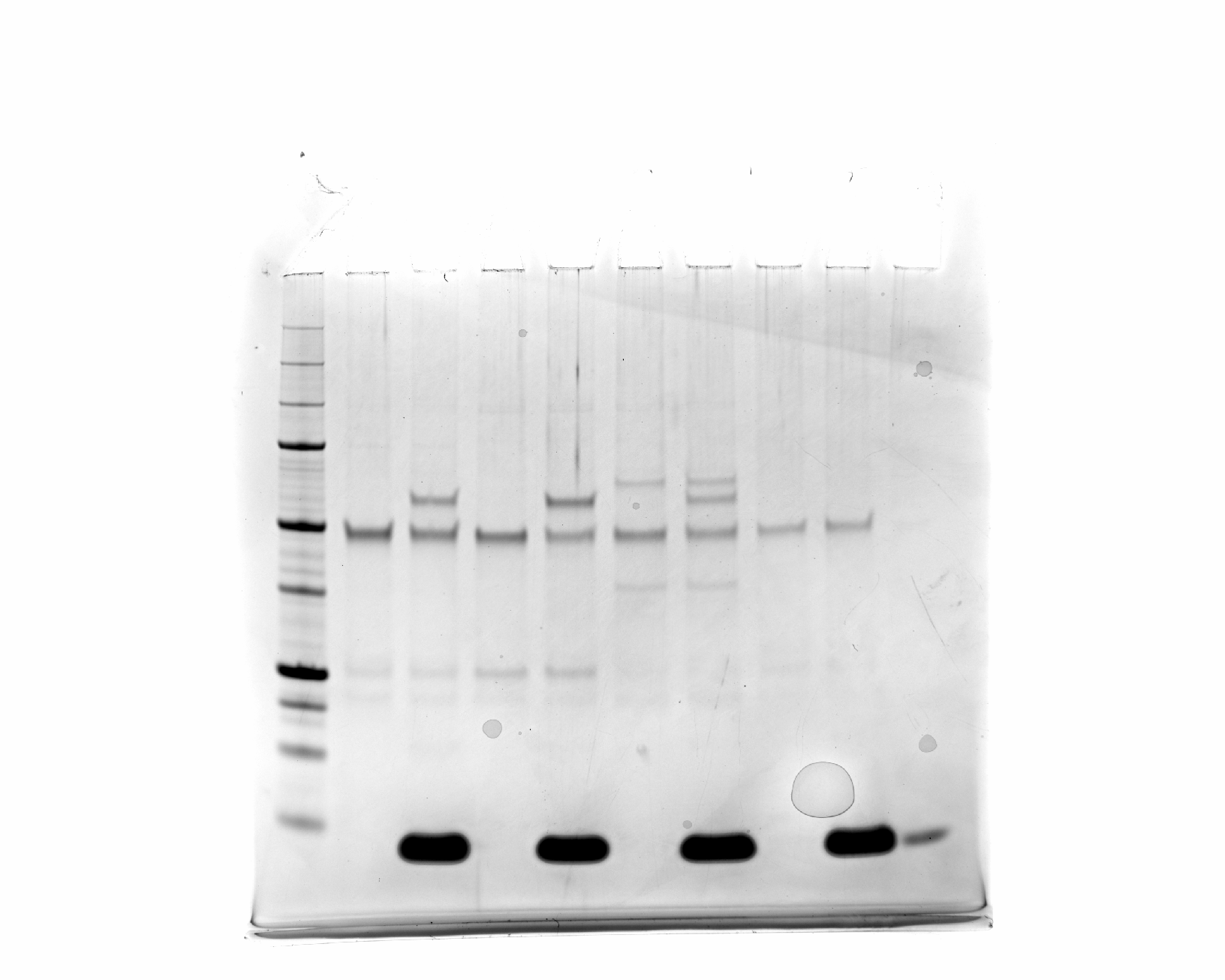

Supplement: Figure 1—figure supplement 1—source data 1. [file elife-90796-fig1-figsupp1-data1.zip › Figure 1-figure supplement 1-source data 1/UbPA assay uncropped_daniel frank 2021-06-01 17h57m01s.tif]

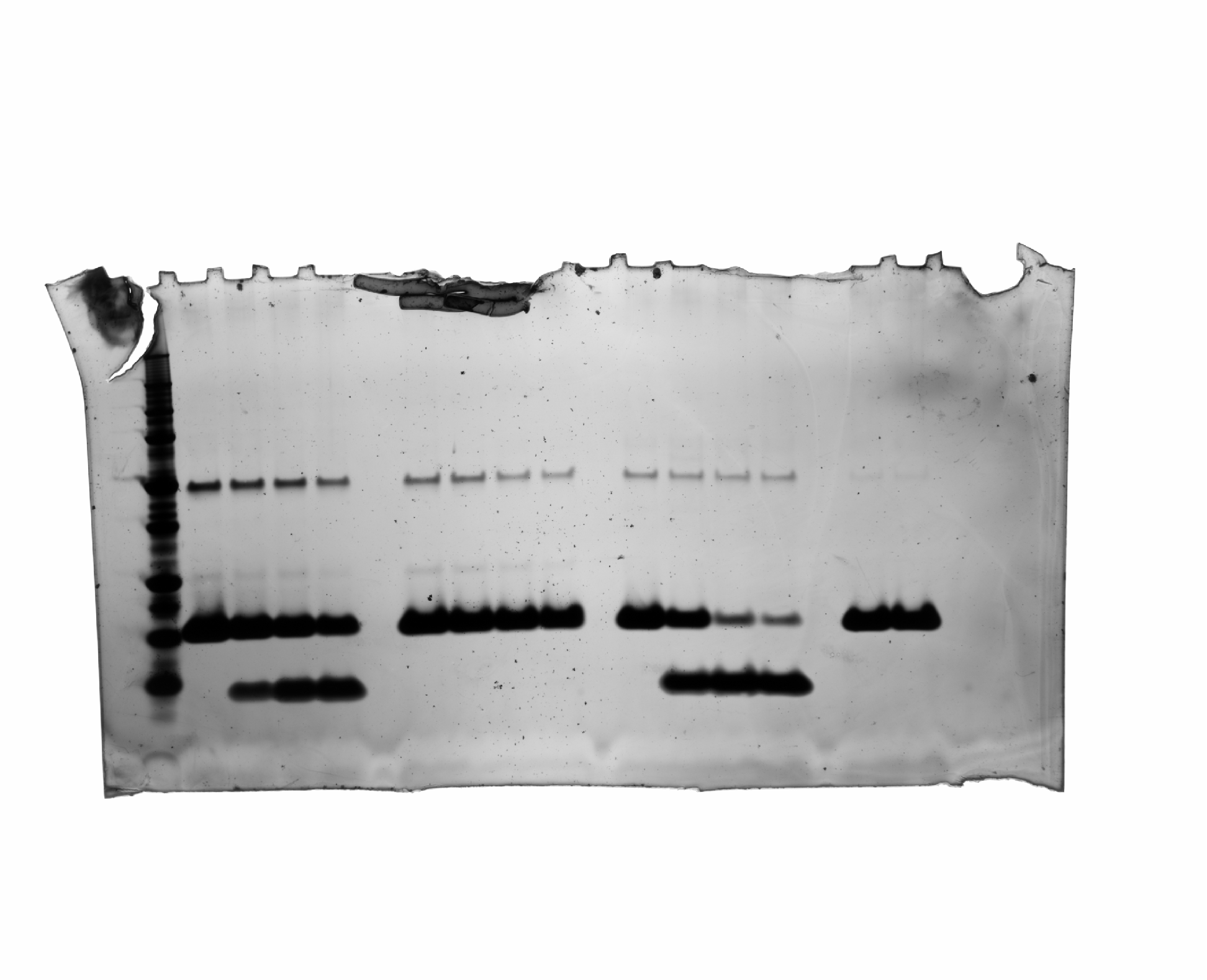

Supplement: Figure 1—figure supplement 2—source data 1. [file elife-90796-fig1-figsupp2-data1.zip › Figure 1-figure supplement 2-source data 1/Fig1 supplement 2 uncropped.tif]

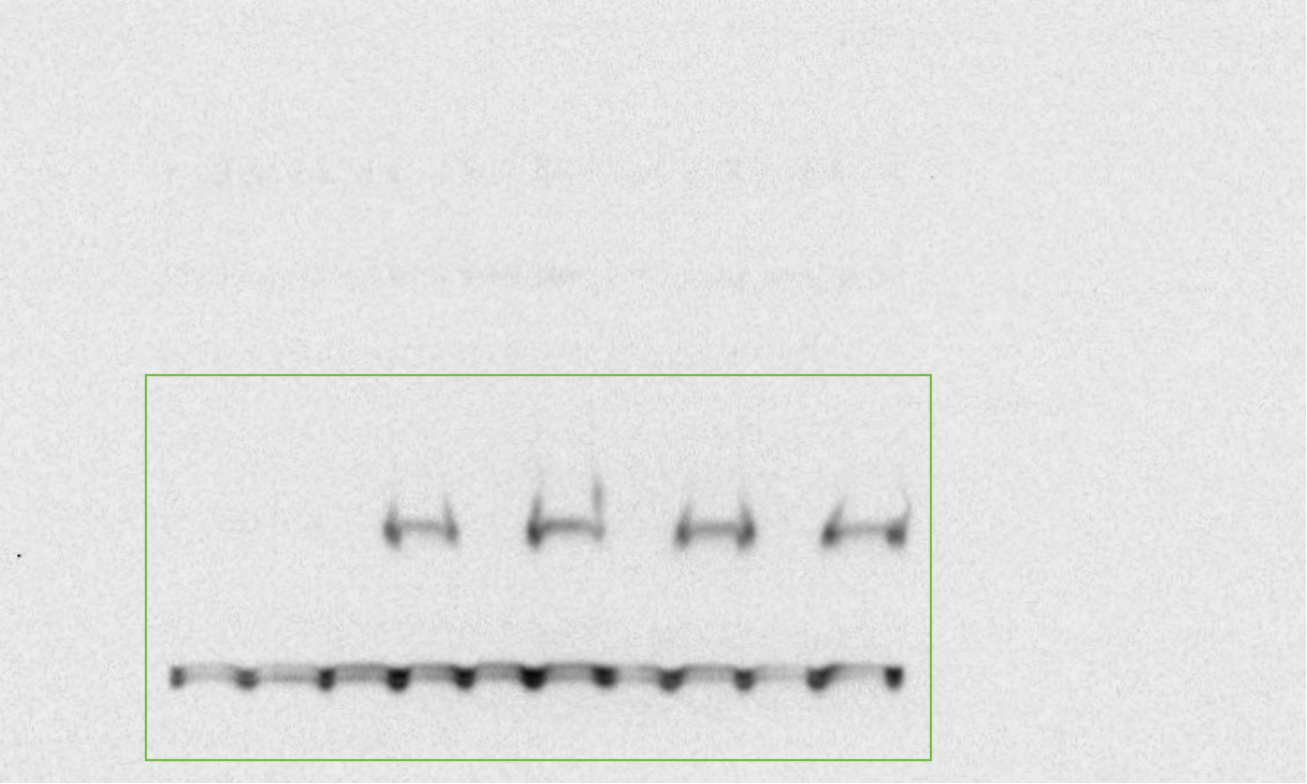

Supplement: Figure 4—source data 1. — Chemiluminescent signals on nitrocellulose blots were acquired using the ChemiDoc Imaging system (Bio-Rad). Included for each of the indicated antibody probe is the uncropped chemiluminescence image with a green box demarcating the region presented in Figure 4. Also included is an image of the chemiluminescence signal merged with an image of the blotting membrane to visualise the positions of pre-stained molecular weight markers (M; HiMark, Invitrogen) relative to the chemiluminescent bands of interest. [file elife-90796-fig4-data1.zip › Figure 4-source data 1/FIG4 inputs wbFLAG+bActin flipped horizontal uncropped_L5W 2019-09-03 16h05m50s.tif]

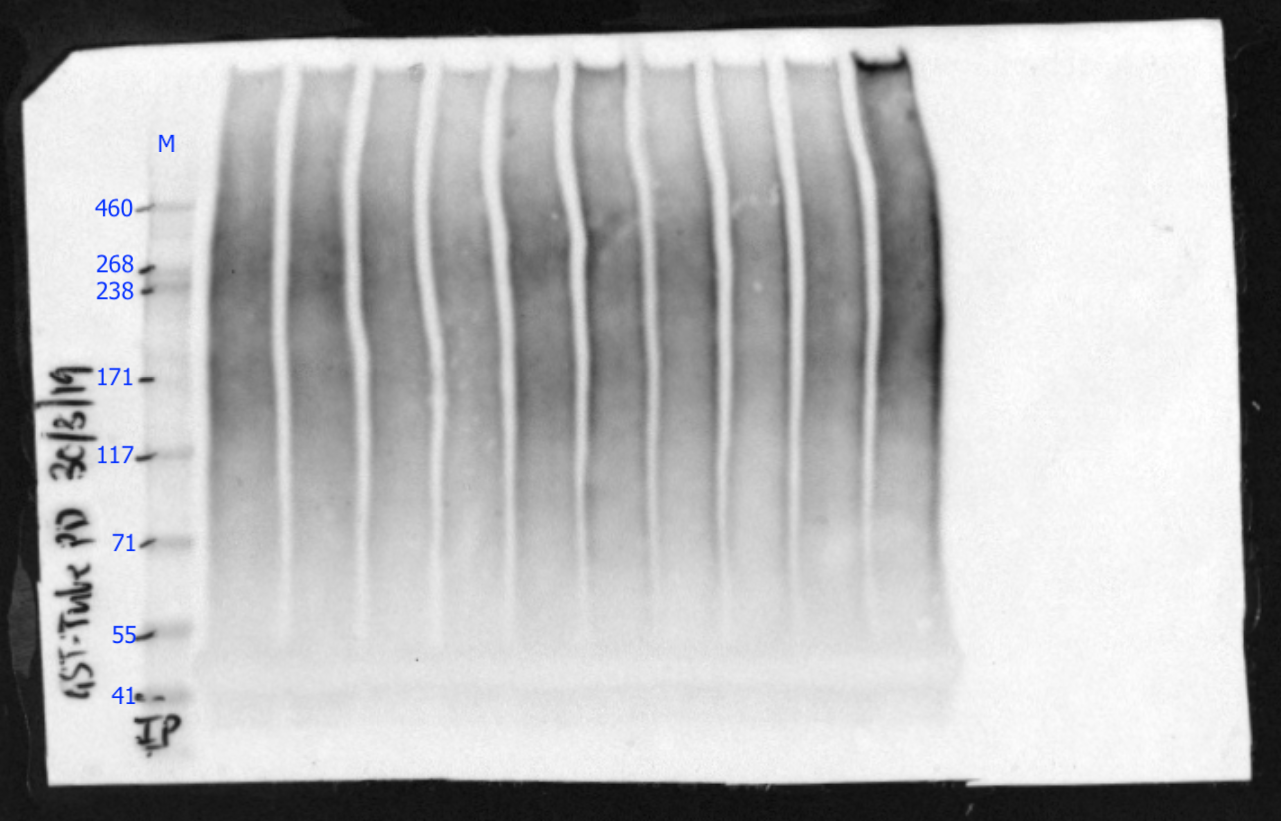

Supplement: Figure 4—source data 1. — Chemiluminescent signals on nitrocellulose blots were acquired using the ChemiDoc Imaging system (Bio-Rad). Included for each of the indicated antibody probe is the uncropped chemiluminescence image with a green box demarcating the region presented in Figure 4. Also included is an image of the chemiluminescence signal merged with an image of the blotting membrane to visualise the positions of pre-stained molecular weight markers (M; HiMark, Invitrogen) relative to the chemiluminescent bands of interest. [file elife-90796-fig4-data1.zip › Figure 4-source data 1/FIG4 TUBE PD wbUbiquitin+membrane_L5W 2019-09-03 16h01m49s+L5W 2019-09-03 16h07m01s.tif]

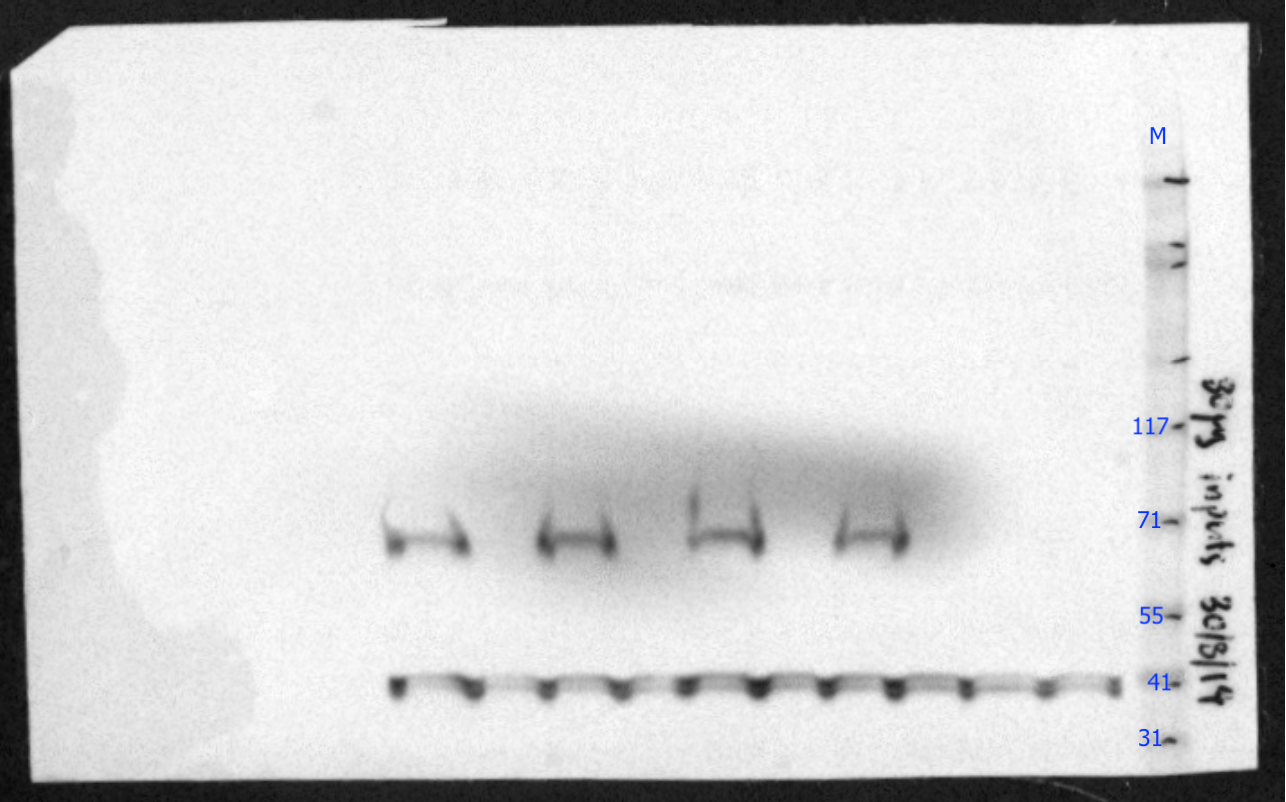

Supplement: Figure 4—source data 1. — Chemiluminescent signals on nitrocellulose blots were acquired using the ChemiDoc Imaging system (Bio-Rad). Included for each of the indicated antibody probe is the uncropped chemiluminescence image with a green box demarcating the region presented in Figure 4. Also included is an image of the chemiluminescence signal merged with an image of the blotting membrane to visualise the positions of pre-stained molecular weight markers (M; HiMark, Invitrogen) relative to the chemiluminescent bands of interest. [file elife-90796-fig4-data1.zip › Figure 4-source data 1/FIG4 inputs wbFLAG+bActin+membrane_L5W 2019-09-03 16h05m50s+L5W 2019-09-03 16h07m01s.tif]

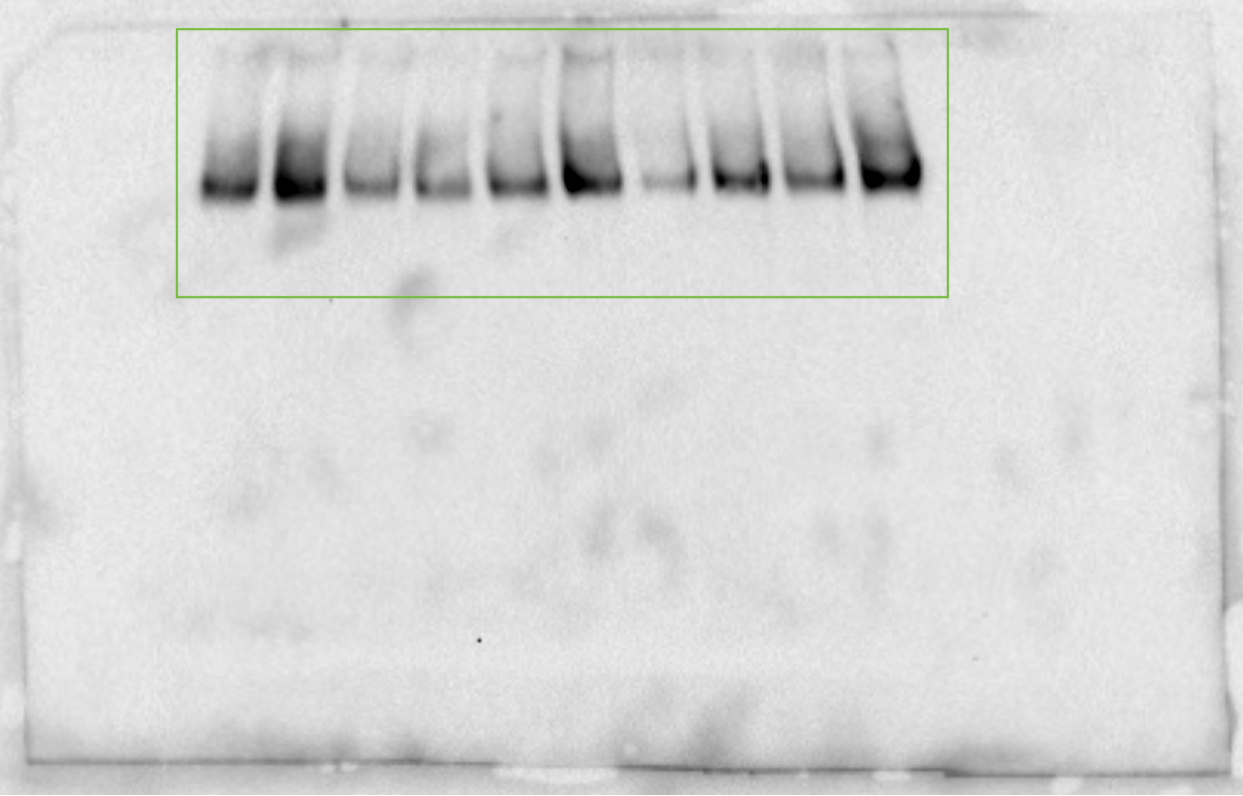

Supplement: Figure 4—source data 1. — Chemiluminescent signals on nitrocellulose blots were acquired using the ChemiDoc Imaging system (Bio-Rad). Included for each of the indicated antibody probe is the uncropped chemiluminescence image with a green box demarcating the region presented in Figure 4. Also included is an image of the chemiluminescence signal merged with an image of the blotting membrane to visualise the positions of pre-stained molecular weight markers (M; HiMark, Invitrogen) relative to the chemiluminescent bands of interest. [file elife-90796-fig4-data1.zip › Figure 4-source data 1/FIG4 TUBE PD wbAPC uncropped_L5W 2019-09-02 11h44m01s.tif]

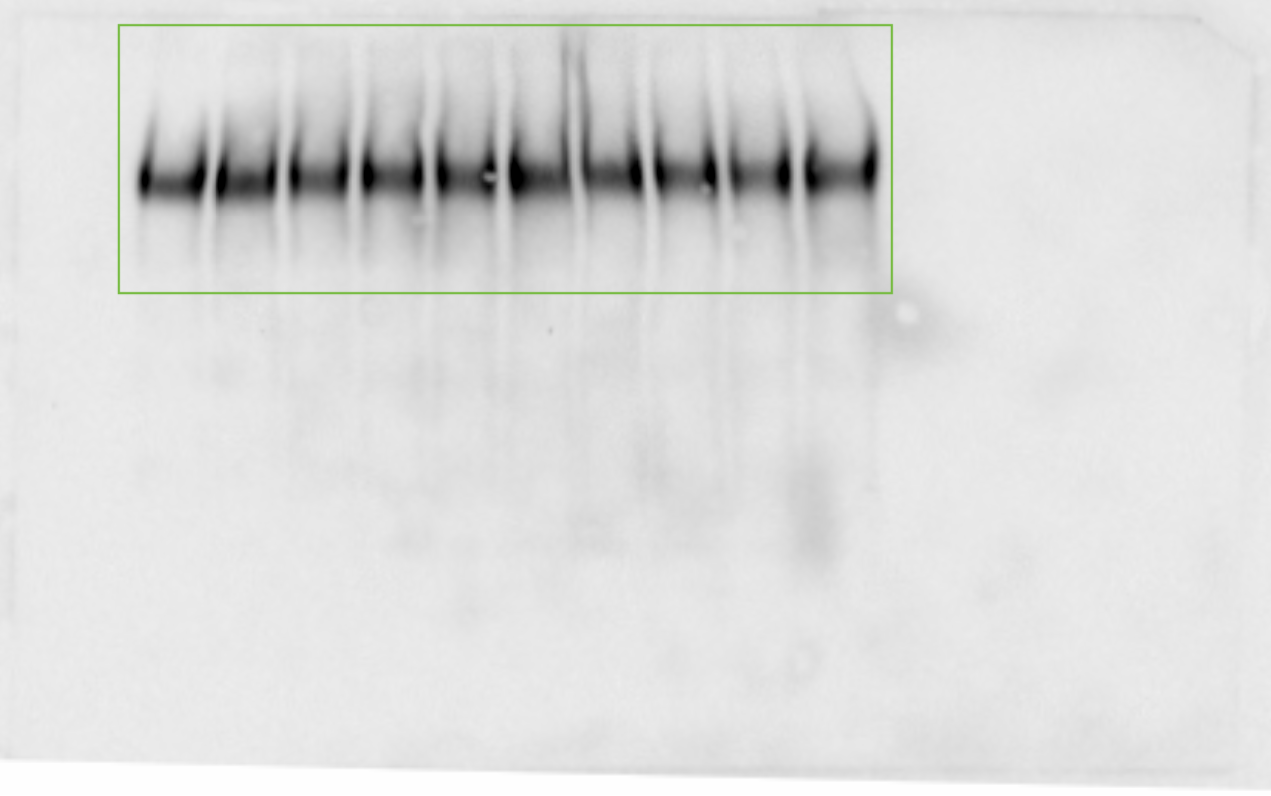

Supplement: Figure 4—source data 1. — Chemiluminescent signals on nitrocellulose blots were acquired using the ChemiDoc Imaging system (Bio-Rad). Included for each of the indicated antibody probe is the uncropped chemiluminescence image with a green box demarcating the region presented in Figure 4. Also included is an image of the chemiluminescence signal merged with an image of the blotting membrane to visualise the positions of pre-stained molecular weight markers (M; HiMark, Invitrogen) relative to the chemiluminescent bands of interest. [file elife-90796-fig4-data1.zip › Figure 4-source data 1/FIG4 inputs wbAPC_flipped horizontal_ uncropped_L5W 2019-09-02 11h42m44s.tif]

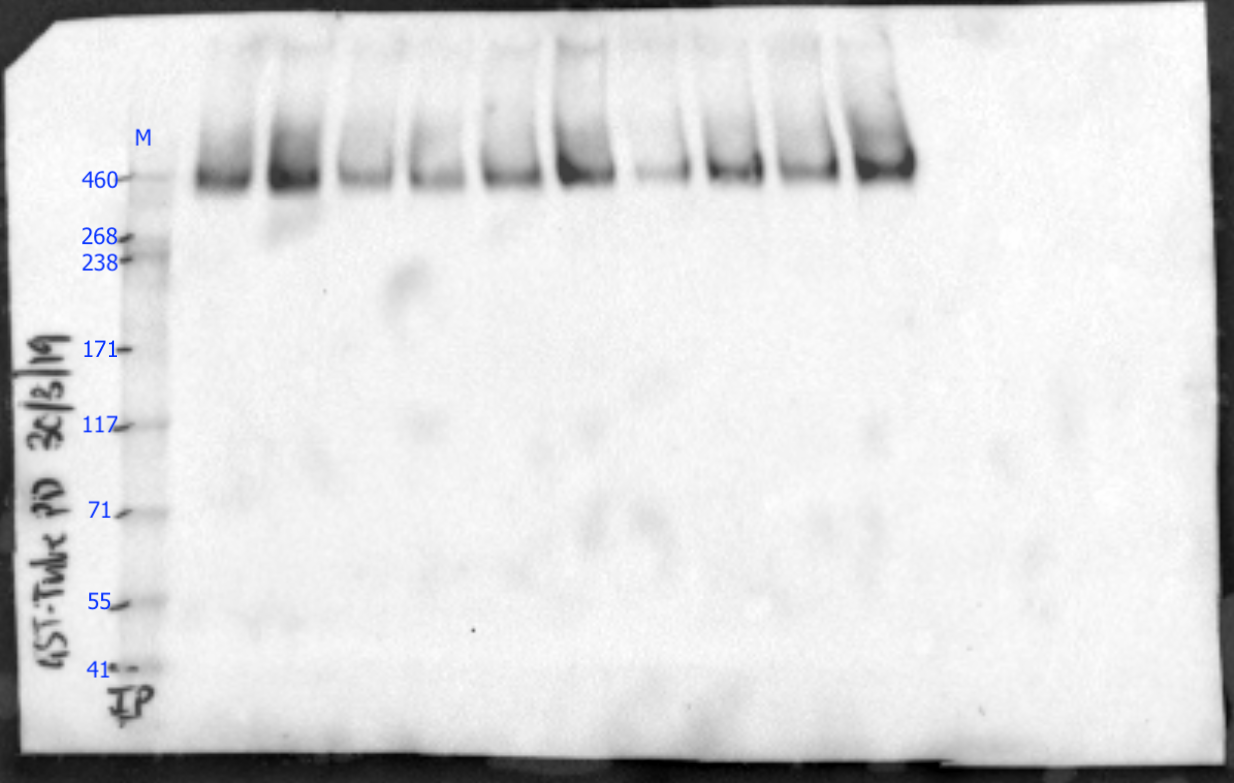

Supplement: Figure 4—source data 1. — Chemiluminescent signals on nitrocellulose blots were acquired using the ChemiDoc Imaging system (Bio-Rad). Included for each of the indicated antibody probe is the uncropped chemiluminescence image with a green box demarcating the region presented in Figure 4. Also included is an image of the chemiluminescence signal merged with an image of the blotting membrane to visualise the positions of pre-stained molecular weight markers (M; HiMark, Invitrogen) relative to the chemiluminescent bands of interest. [file elife-90796-fig4-data1.zip › Figure 4-source data 1/FIG4 TUBE PD wbAPC+membrane_L5W 2019-09-02 11h44m01s+L5W 2019-09-02 11h47m45s.tif]

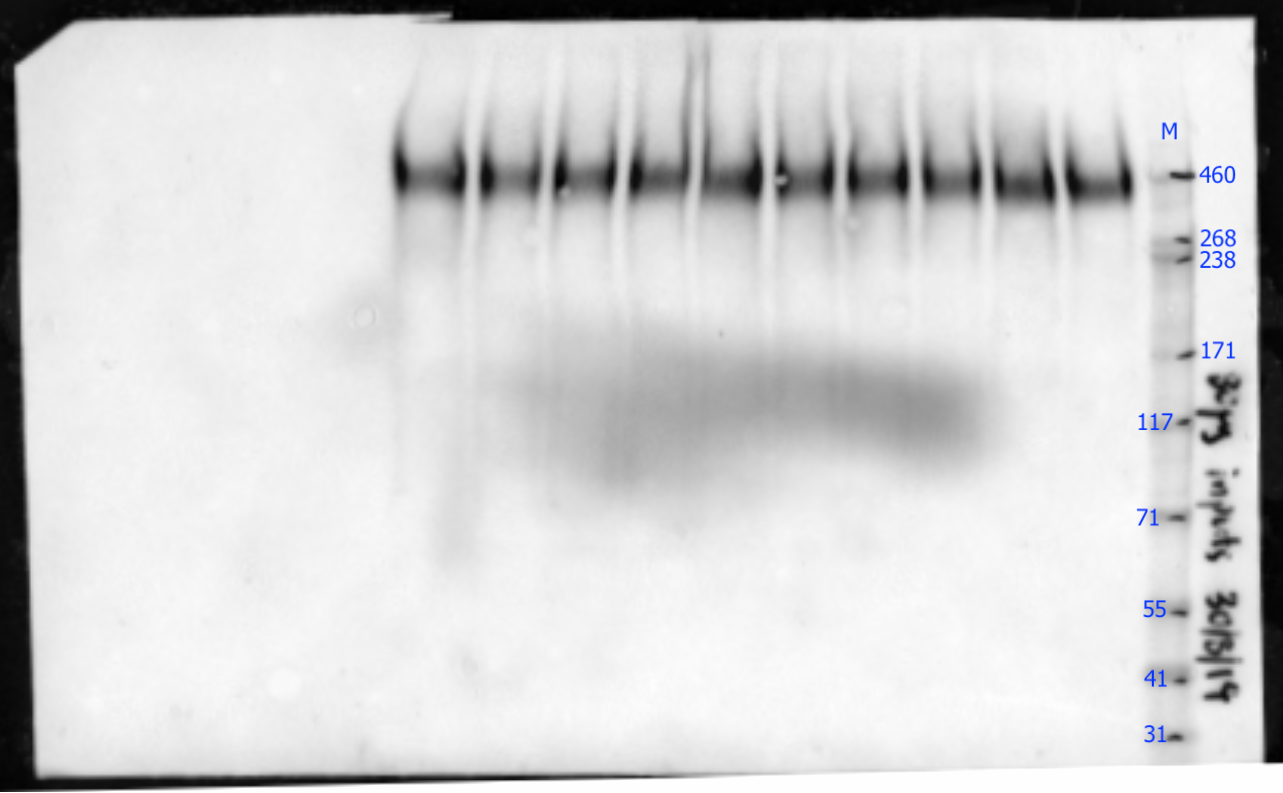

Supplement: Figure 4—source data 1. — Chemiluminescent signals on nitrocellulose blots were acquired using the ChemiDoc Imaging system (Bio-Rad). Included for each of the indicated antibody probe is the uncropped chemiluminescence image with a green box demarcating the region presented in Figure 4. Also included is an image of the chemiluminescence signal merged with an image of the blotting membrane to visualise the positions of pre-stained molecular weight markers (M; HiMark, Invitrogen) relative to the chemiluminescent bands of interest. [file elife-90796-fig4-data1.zip › Figure 4-source data 1/FIG4 inputs wbAPC+membrane_L5W 2019-09-02 11h42m44s+L5W 2019-09-02 11h47m45s.tif]

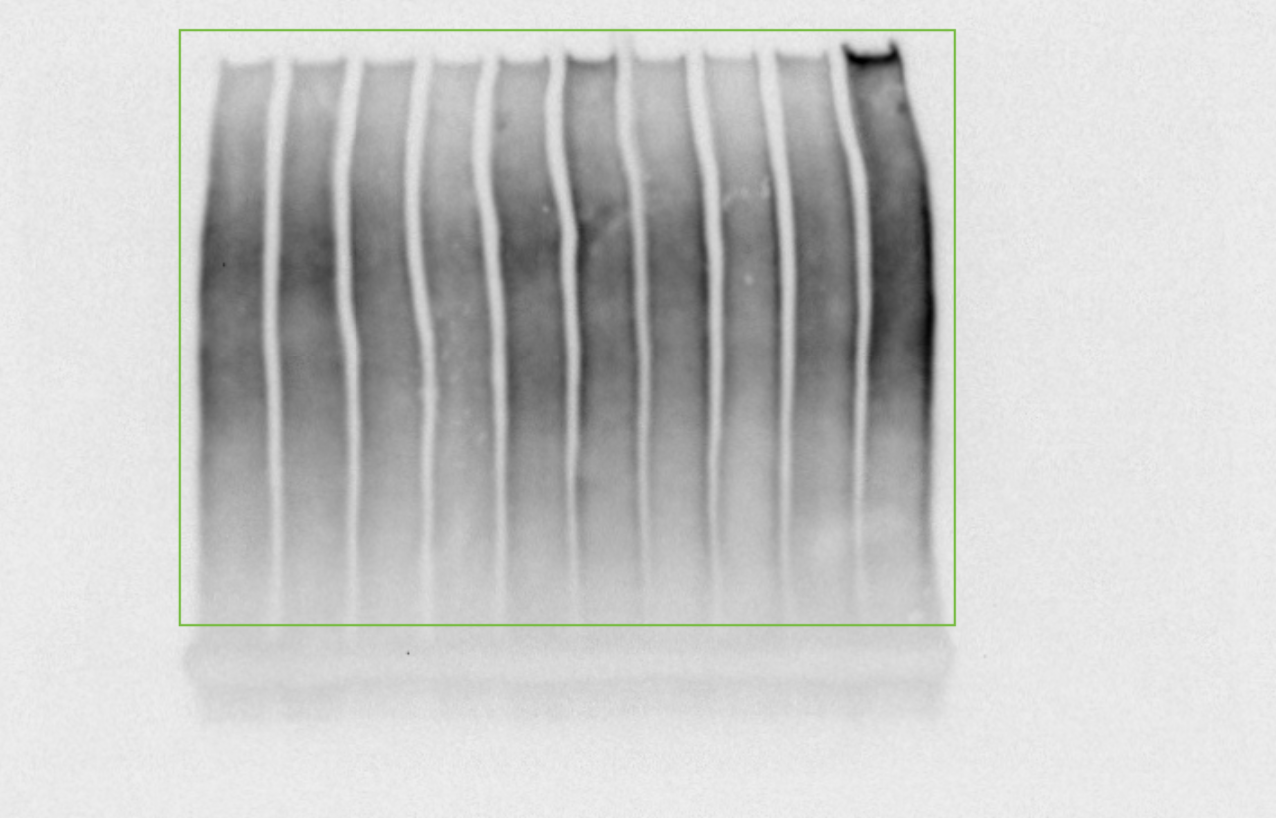

Supplement: Figure 4—source data 1. — Chemiluminescent signals on nitrocellulose blots were acquired using the ChemiDoc Imaging system (Bio-Rad). Included for each of the indicated antibody probe is the uncropped chemiluminescence image with a green box demarcating the region presented in Figure 4. Also included is an image of the chemiluminescence signal merged with an image of the blotting membrane to visualise the positions of pre-stained molecular weight markers (M; HiMark, Invitrogen) relative to the chemiluminescent bands of interest. [file elife-90796-fig4-data1.zip › Figure 4-source data 1/FIG4 TUBE PD wbUbiquitin uncropped_L5W 2019-09-03 16h01m49s.tif]

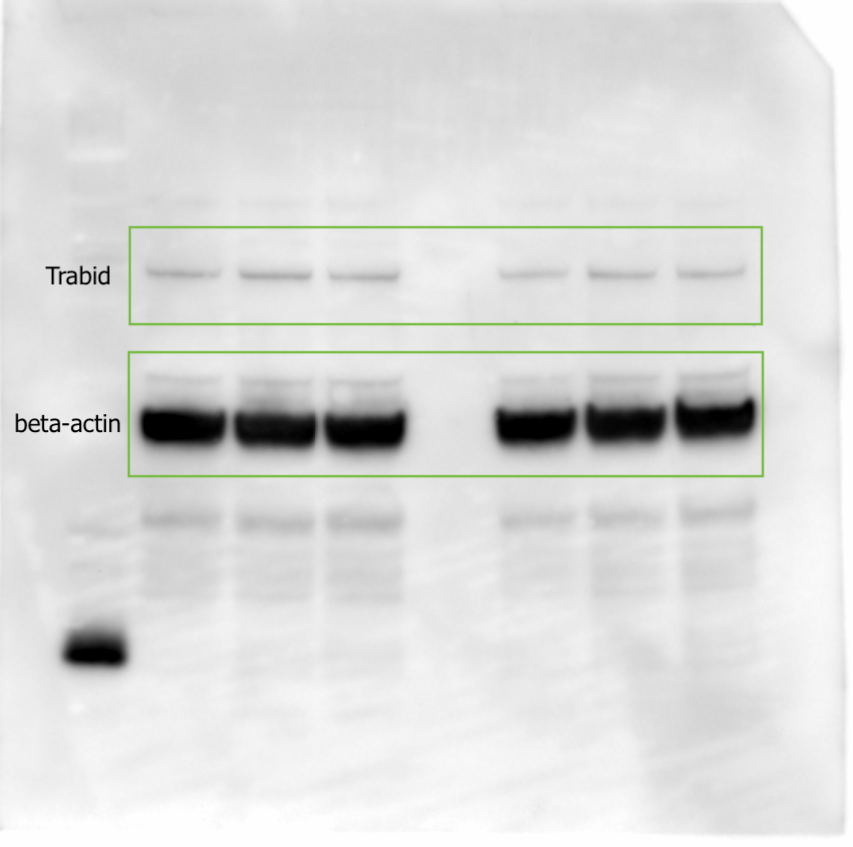

Supplement: Figure 6—source data 1. — Chemiluminescent signals on polyvinylidene fluoride (PVDF) blots were acquired using the ChemiDoc Imaging system (Bio-Rad). Included is the uncropped chemiluminescence image with green boxes demarcating the regions presented in Figure 6B. Also included is the chemiluminescence image merged with an image of the blotting membrane to visualize the positions of pre-stained molecular weight protein standards (M; Novex Sharp, Invitrogen) relative to the chemiluminescent bands of interest. [file elife-90796-fig6-data1.zip › Figure 6-source data 1/FIG6 wbTrabid+b-Actin flipped horizontal_uncropped_hoanh 2023-03-14 13h28m26s.tif]

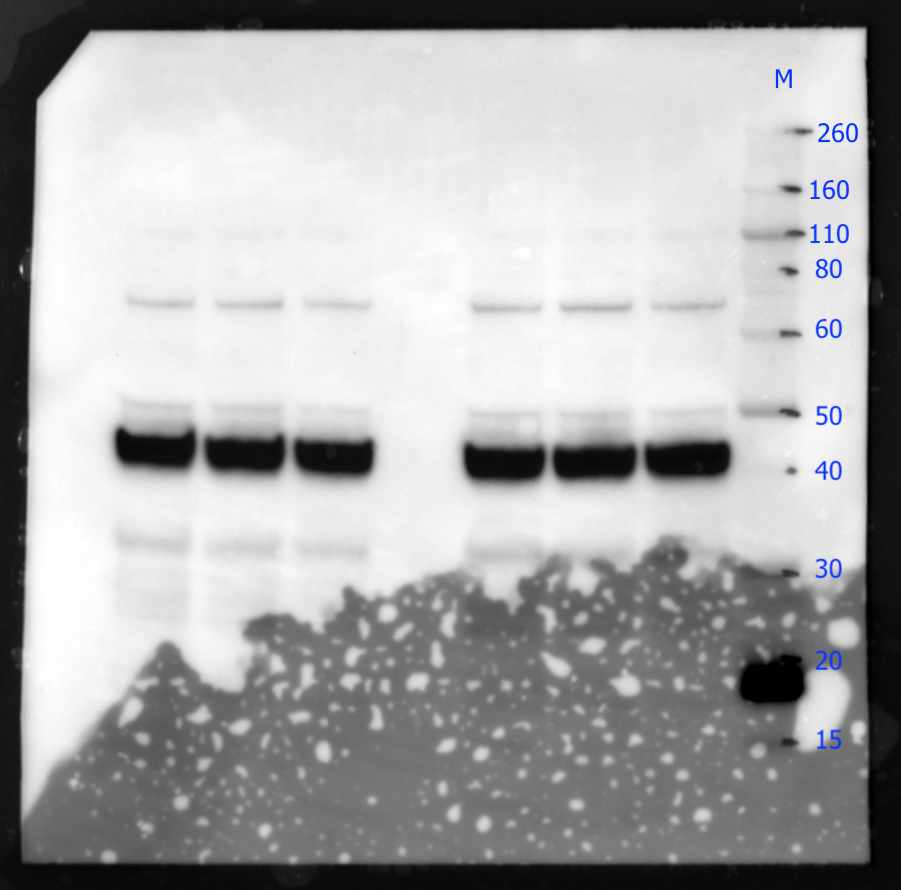

Supplement: Figure 6—source data 1. — Chemiluminescent signals on polyvinylidene fluoride (PVDF) blots were acquired using the ChemiDoc Imaging system (Bio-Rad). Included is the uncropped chemiluminescence image with green boxes demarcating the regions presented in Figure 6B. Also included is the chemiluminescence image merged with an image of the blotting membrane to visualize the positions of pre-stained molecular weight protein standards (M; Novex Sharp, Invitrogen) relative to the chemiluminescent bands of interest. [file elife-90796-fig6-data1.zip › Figure 6-source data 1/FIG6 wbTrabid+bActin+membrane_hoanh 2023-03-14 13h28m26s+hoanh 2023-03-14 13h31m04s.tif]
